# Identification of *TMPRSS3* as a Significant Contributor to Autosomal Recessive Hearing Loss in the Chinese Population

**DOI:** 10.1155/2017/3192090

**Published:** 2017-06-13

**Authors:** Xue Gao, Sha-Sha Huang, Yong-Yi Yuan, Jin-Cao Xu, Ping Gu, Dan Bai, Dong-Yang Kang, Ming-Yu Han, Guo-Jian Wang, Mei-Guang Zhang, Jia Li, Pu Dai

**Affiliations:** ^1^Department of Otolaryngology, Head and Neck Surgery, PLA General Hospital, No. 28, Fuxing Road, Beijing 100853, China; ^2^Department of Otolaryngology, The General Hospital of the PLA Rocket Force, No. 16, XinWai Da Jie, Beijing 100088, China; ^3^Department of Otorhinolaryngology, Shenzhen Children's Hospital, No. 7019, Yitian Road, Shenzhen 518026, China; ^4^Department of Otolaryngology, Xi'an Medical College, No. 1, XinWang Road, Wei yang qu, Xi'an 710021, China

## Abstract

Hereditary hearing loss is characterized by a high degree of genetic heterogeneity. Mutations in the *TMPRSS3* (transmembrane protease, serine 3) gene cause prelingual (DFNB10) or postlingual (DFNB8) deafness. In our previous study, three pathogenic mutations in *TMPRSS3* were identified in one Chinese family. To evaluate the importance of *TMPRSS3* mutations in recessive deafness among the Chinese, we screened 150 autosomal recessive nonsyndromic hearing loss (ARNSHL) families and identified 6 that carried seven causative *TMPRSS3* mutations, including five novel mutations (c.809T>A, c.1151T>G, c.1204G>A, c.1244T>C, and c.1250G>A) and two previously reported mutations (c.323-6G>A and c.916G>A). Each of the five novel mutations was classified as severe, by both age of onset and severity of hearing loss. Together with our previous study, six families were found to share one pathogenic mutation (c.916G>A, p.Ala306Thr). To determine whether this mutation arose from a common ancestor, we analyzed six short tandem repeat (STR) markers spanning the *TMPRSS3* gene. In four families, we observed linkage disequilibrium between p.Ala306Thr and STR markers. Our results indicate that mutations in *TMPRSS3* account for about 4.6% (7/151) of Chinese ARNSHL cases lacking mutations in *SLC26A4* or *GJB2* and that the recurrent *TMPRSS3* mutation p.Ala306Thr is likely to be a founder mutation.

## 1. Introduction

Hearing impairment is a very common sensory disorder, affecting 1 of 500–650 newborns [1, 2]. Genetic factors contribute to approximately 60% of congenital sensorineural hearing loss cases. Nonsyndromic hearing loss (NSHL), in which hearing impairment is the only obvious clinical abnormality, accounts for 70% of genetic cases. To date, more than 200 genetic loci have been mapped, and 100 deafness genes have been identified (http://hereditaryhearingloss.org/). Autosomal recessive nonsyndromic hearing loss (ARNSHL) is the most common type and accounts for ~80% of cases of inherited hearing loss.

Individuals with mutations in *TMPRSS3* (transmembrane protease, serine 3) present with two different phenotypes: DFNB10-associated hearing impairment that is prelingual (OMIM 605511) and DFNB8-associated hearing impairment that is typically late onset and postlingual (OMIM 601072). *TMPRSS3* mutations can be divided into mild or severe, and the hearing phenotype is dependent upon the combination of two *TMPRSS3* mutations. The combination of two severe mutations leads to prelingual, profound hearing loss, whereas severe mutations in combination with mild mutations lead to a milder phenotype with postlingual-onset hearing loss [3, 4]. The genetic load of *TMPRSS3* in ARNSHL varies with ethnicity but is commonly a responsible gene in several populations. The frequency of *TMPRSS3* mutations in childhood ARNSHL cases was 12% (3/25) in Turkish families negative for *GJB2* mutations [5]; 13.1% (5/38) in Slovenian ARNSHL patients negative for *GJB2*, *GJB6*, and mitochondrial A1555G mutations [6]; and 0.45% (2/448) in a European population with childhood deafness negative for the *GJB2* 35delG mutation [7]. The frequency is approximately 1.8% (8/449) in the Pakistani population [8], 5% (2/39) in Tunisian families affected by profound ARNSHL [9], 2.5% (1/40) in a Korean ARNSHL study [10], and 5.9% (3/51) in a Korean ARNSHL population negative for the *GJB2* mutation [11]. In some populations, *GJB2* mutations were not excluded and only congenital and profound hearing loss cases were involved. In these populations, *TMPRSS3* mutations might still be a significant cause of deafness.

Although several causative mutations in *TMPRSS3* have been identified, little is known about the contribution of this gene to ARNSHL in the Chinese population. In a prior study, we performed targeted next-generation sequencing of 129 known deafness genes in one Chinese ARNSHL family (FH1523) and identified 3 *TMPRSS3* mutations (c.36delC, c.316C>T, and c.916G>A) [12]. In this study, we screened the *TMPRSS3* coding region in 150 ARNSHL families previously shown to lack mutations in *GJB2* or *SLC26A4* genes. In seven families, we identified two known and five novel mutations in *TMPRSS3*. A previously reported mutation (c.916G>A, p.Ala306Thr) is identified as founder mutation in four families. Our results suggest that mutations in *TMPRSS3* are a relatively common cause for ARNSHL in the Chinese population.

## 2. Materials and Methods

### 2.1. Ethics Statement

This study was approved by the Chinese PLA General Hospital Research Ethics Committee (Beijing, China). Fully informed written consent for participation and for publication of clinical data was obtained from each subject or from the guardians of subjects < 18 years old (yo).

### 2.2. Clinical Data

DNA samples from the Departments of Otolaryngology and of Head and Neck Surgery, PLA General Hospital, were analyzed. The 150 affected patients originated from 150 families presenting with ARNSHL, in whom previous screening had found no mutations within the *GJB2* or *SLC26A4* genes. Computed tomography of the temporal bone was performed on the index patients of each family. A physical examination, otoscopy, and pure-tone audiometric examination (at frequencies from 125 to 8000 Hz) were performed to establish the diagnosis of sensorineural hearing loss. The hearing loss range was described based on pure-tone audiometry (PTA) parameters: low frequency, 125–500 Hz; mid frequency, 1-2 kHz; and high frequency, 4–8 kHz. Prelingual and postlingual hearing loss were classified by the onset age of prominent hearing loss. Prelingual hearing loss is present before speech develops and usually begins before age 3, whereas postlingual hearing loss occurs after the development of normal speech [13]. Evaluation of vestibular function included evaluation of the vestibulo-ocular reflex using electronystagmography with computer analysis and saccadic, smooth-pursuit, and horizontal optokinetic nystagmus responses. Vestibular stimulation comprised rotatory and caloric tests.

### 2.3. DNA Preparation

All of the genomic DNA was extracted from peripheral blood using a blood DNA extraction kit, according to the manufacturer's instructions (TianGen, Beijing, China).

### 2.4. Mutational Detection and Analysis of *TMPRSS3*

All of the 13 exons and 100 bp of exon-intron boundaries of *TMPRSS3* (NM_024022.2) were screened via Sanger sequencing of DNA from 150 index patients of ARNSHL families. Primer sequences are available upon request. To identify pathogenic mutations, cosegregation analyses were performed with the family members and with an in-house database of 481 Chinese controls with normal hearing.

### 2.5. Multiple Sequence Alignment

Multiple sequence alignment was performed for the five novel mutations identified, using a HomoloGene package with default settings and the sequences *NP_001243246*.*1* (*H. sapiens*), *XP_001105841*.*2* (*M. mulatta*), *XP_001137100*.*3* (*P. troglodytes*), *XP_001179855*.*1* (*B. taurus*), *XP_853682*.*3* (*C. lupus*), *NP_001157248*.*1* (*M. musculus*), *NP_001101089*.*1* (*R. norvegicus*), *XP_425558*.*3* (*G. gallus*), and *XP_001340422*.*5* (*D. rerio*) (http://www.ncbi.nlm.nih.gov/homologene?cmd=Retrieve&dopt=MultipleAlignment&list_uids=56985).

### 2.6. Haplotype Analysis

DNA samples (23) from six families were haplotyped using six STR markers (D21S266, D21S1260, D21S2092, D21S1225, D21S1411, and D21S1890) within a 1 Mb region surrounding *TMPRSS3* Ala306, as previously described (Table 1) [11]. Haplotype analysis was performed by direct sequencing.

## 3. Results

### 3.1. Mutation Analysis of *TMPRSS3*

**Figure 1:**
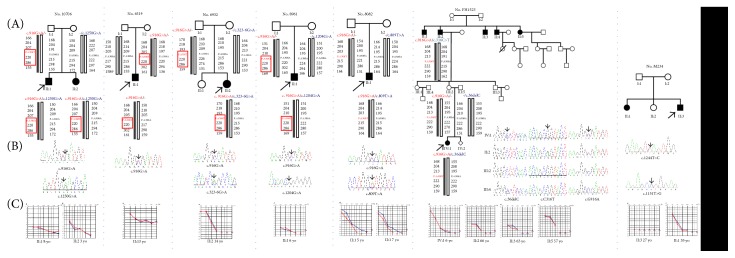
Pedigree, haplotype analysis, audiogram, and mutational analysis of families with *TMPRSS3* mutations. (A) Affected subjects are denoted in black. The proband is indicated by an arrow. Haplotype analysis in six families with the recurrent mutation TMPRSS3 p.Ala306Thr. Haplotypes are shown with the linked haplotype in boxes. A recombination event between p.Ala306Thr and D21S1225 was observed in four families while recombination event between p.Ala306Thr and D21S1411 was observed in two families. (B) DNA sequencing profile. (C) Audiograms of the affected subjects. Hearing loss appears to be progressive (red, right ear; blue, left ear).

**Figure 2:**
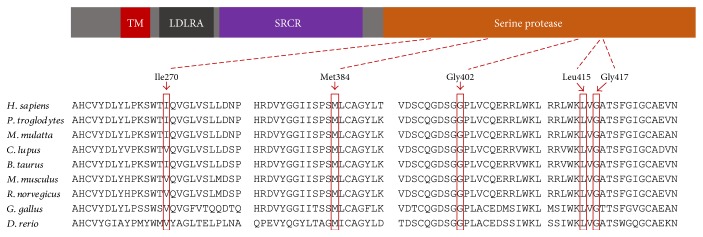
Conservation analysis of *TMPRSS3* mutations and genomic structure of *TMPRSS3* based on the open reading frame (NM_024022.2). Protein alignment showing conservation of residues TMPRSS3 Ile270, Met384, Gly402, Leu415, and Gly417 across nine species. All mutations occurred at evolutionarily conserved amino acids or areas (in red box) in trypsin-like serine protease domain. TM: transmembrane domain; LDLRA: LDL receptor-like domain; SRCR: scavenger receptor cysteine-rich domain; serine protease: trypsin-like serine protease domain.

In our previous study of one Chinese ARNSHL family (FH1523), three disease-segregating mutations in *TMPRSS3* (c.916G>A, p.Ala306Thr; c.316C>T, p.Arg106Cys; and c.36delC, p.Pro12fs) were identified and described [12]. In this study, we performed direct sequencing of *TMPRSS3* in probands of another 150 Chinese ARNSHL families negative for *GJB2* and *SLC26A4* mutations. Among these, we identified six families carrying seven causative *TMPRSS3* mutations, including five novel mutations (c.809T>A, p.Ile270Asn; c.1151T>G, p.Met384Arg; c.1204G>A, p.Gly402Arg; c.1244T>C, p.Leu415Ser; and c.1250G>A, p.Gly417Glu) and two previously reported pathogenic mutations (c.916G>A, p.Ala306Thr and c.323-6G>A) (Figure 1) [4, 8, 14]. The five novel mutations were all located within the catalytic serine protease domain. Analysis using Polyphen-2 software predicted them to be damaging, and they were also identified as deleterious by analysis using SIFT. These amino acid substitutions occurred in an evolutionarily conserved region (Figure 2). They had not been reported in previous studies, were not present in the ExAC database (http://exac.broadinstitute.org/), and were not seen in the 481 Chinese controls with normal hearing. Combining the results of this and our previous study, the frequency of *TMPRSS3* mutations found in Chinese ARNSHL families was 4.6% (7/151). The most prevalent mutation was c.916G>A (p.Ala306Thr) at 2% (6/302), accounting for 47% of all *TMPRSS3* mutations.

### 3.2. Haplotype Analysis

To determine whether the c.916G>A (p.Ala306Thr) mutation found in families FH1523, 6932, 8082, 8961, 6519, and 10706 was derived from a common founder, we performed linkage analysis using 6 STR markers in 23 DNA samples (Figure 1, (A)). In four families, we observed linkage disequilibrium between p.Ala306Thr and one STR marker (D21S1260) within a 97 kb interval, while in two of the four families, we found linkage disequilibrium between p.Ala306Thr and two STR markers (D21S1260 and D21S266) within a 359 kb interval, suggesting that p.Ala306Thr probably arose from a common founder, as was previously reported in a Korean population [11].

### 3.3. Clinical Characteristics and Genotype-Phenotype Correlations

Table 2 summarizes clinical characteristics of 14 patients from seven ARNSHL families with *TMPRSS3* mutations including onset age, audiogram configuration, progression of hearing loss, and vestibular symptoms. Age at onset ranged from newborn to 40 years, although the majority of patients showed evidence of deafness in childhood, at 3 to 6 years of age. None of the patients had vestibular symptoms. Examining the types of hearing loss identified some correlations between genotype and phenotype (Figure 1(B and C)). Hearing loss in 10706-II:1 (male/8 yo) and 10706-II:2 (female/3 yo) (Figure 1) was diagnosed at 2 yo, and their hearing loss progressed rapidly with age. An audiogram of 10706-II:1 at 8 yo was flat with an average PTA of 90-decibel hearing level (dB HL), whereas that of 10706-II:2 at 3 yo showed ski-slope loss with an average PTA of 85 dB HL. 10706-II:2 underwent cochlear implantation at age 3, and her language ability improved after surgery. The proband and his affected sister were compound heterozygotes for c.916G>A (p.Ala306Thr) and one novel mutation c.1250G>A (p.Gly417Glu). In family 6519 (Figure 1), c.916G>A (p.Ala306Thr) was detected in patient II:1 in the heterozygous state. The patient's mother with normal hearing was also heterozygous for the mutation. No other candidate mutation in the coding region of *TMRPSS3* was detected. Hearing loss in 6519-II:1 (Figure 1, male/3 yo) was initially detected at age 3, with a ski-slope audiogram and a normal threshold at 250 Hz; the average threshold at 500 Hz to 4 kHz was 55 dB HL. We speculated that a gene copy number variation might exist, that a mutation may lie within a noncoding region, or that other deafness genes were responsible. Hearing loss in 6932-II:1 (Figure 1, female/17 yo) was initially detected by the age of 9 yo with a ski-slope audiogram. The threshold at 125 and 250 Hz was 10 dB HL and that at 2 Hz to 8 kHz was greater than 100 dB HL. The patient complained of slowly progressing hearing loss with age. She underwent cochlear implantation at age 14, and her language ability improved after surgery. Patient 6932-II:1 was a compound heterozygote for c.916G>A (p.Ala306Thr) and c.323-6G>A; both mutations were previously reported. The splice site mutation c.323-6G>A was reported to be pathogenic in Dutch and Pakistani patients [3, 8]. However, there was inconsistency in this classification for the c.323-6G>A (p.Cys107fs) mutation. In the Dutch family, the c.323-6G>A mutation was (relatively) severe and homozygous mutation results in prelingual (DFNB10) hearing impairment. However, a homozygous c.323-6G>A mutation was described by Veske et al. (1996) to be the underlying cause of postlingual (DFNB8) hearing impairment. In this study, considering the hearing phenotype of 6932-II:1, we classified c.323-6G>A as a mild mutation.

Hearing loss in 8082-II:1 (Figure 1, female/5 yo) was initially detected at 5 yo with a ski-slope audiogram. Her parents had noticed occasional poor hearing since she was 3 yo. At age 5, she showed severe hearing loss at 1–8 kHz and moderate hearing loss at 250–500 Hz, with a normal threshold at 125 Hz. Thereafter, the hearing loss showed slow progression, with annual threshold deteriorations of 5–8 dB HL at 500 Hz and 2.5–5 dB HL at 1–8 kHz, according to a recent audiogram at 7 yo. She is now wearing hearing aids. Patient 8082-II:1 was a compound heterozygote for c.916G>A (p.Ala306Thr) and the novel mutation c.809T>A (p.Ile270Asn). The novel missense mutation c.809T>A is located within exon 9 and causes an amino acid substitution from isoleucine to asparagine at position 270, which is close to the active site histidine (H257), and therefore affects the activity of the enzyme. Hearing loss of 8961-II:1 (Figure 1, male/6 yo) was initially detected by the age of 2, with the auditory brainstem response (ABR) threshold for both ears significantly elevated to 97 dB HL. According to the parent's description, hearing loss progressed slowly. A recent audiogram at 6 yo was flat, and the average PTA was more than 90 dB HL. Patient 8961-II:1 was a compound heterozygote for c.916G>A (p.Ala306Thr) and the novel mutation c.1204G>A (p.Gly402Arg). Hearing loss in M234-II:1 (female/35 yo) and M234-II:3 (male/27 yo) (Figure 1) was detected at 3 yo, and their hearing loss progressed rapidly with advancing age. An audiogram of M234-II:3 at 27 yo was flat, and the average PTA was more than 90 dB HL, while that of M234-II:1 at 35 yo was a ski-slope, with an average PTA of 75 dB HL. The two patients carried two of the novel mutations as compound heterozygotes: c.1151T>G (p.Met384Arg) and c.1244T>C (p.Leu415Ser). Family FH1523 has previously been described [12]. According to the onset and severity of hearing loss, the five novel missense mutations were classified as severe mutations, while c.323-6G>A was probably a mild mutation.

## 4. Discussion

The function of TMPRSS3 is very important to the auditory system; it has also been identified as a tumor-associated gene that is overexpressed in pancreatic, ovarian, and breast tumors [15–17]. In 2001, Scott et al. showed that *TMPRSS3* was mutated in nonsyndromic autosomal recessive deafness (DFNB8/10) and is associated with both congenital and childhood-onset forms [14]. TMPRSS3 contains a transmembrane domain, a low-density lipoprotein receptor class A domain, a scavenger receptor cysteine-rich domain, and a trypsin-like serine protease domain (NP_001243246.1). TMPRSS3 is expressed in spiral ganglion neurons, inner hair cells, supporting cells, and stria vascularis of the rat cochlea [18]. It plays an important role in activating the ENaC sodium channel, which is regulated by serine protease activity [19], and maintains a low Na^+^ concentration in the endolymph of the inner ear [3, 18, 20].

The typical ski-slope audiogram configuration in ARNSHL is suggestive of *TMPRSS3* involvement, with a hearing phenotype and inheritance similar to those of *SLC26A4*. Our results indicate that *TMPRSS3* mutations account for about 4.6% (7/151) of ARNSHL in Chinese patients negative for *GJB2* and *SLC26A4* mutations, an incidence similar to that seen in Korean and Tunisian populations [9, 11]. It has been reported that mutations in different domains of TMPRSS3 result in various hearing impairment phenotypes, likely due to the distinct influence on protease activity of different mutations [3, 5, 7–11, 14, 18, 21–29]. Lee et al. proposed that disruption of the proteolytic activity of TMPRSS3 is tightly correlated with the pathogenesis of hearing loss and predicted that mutations in the SRCR and LDLRA domains affect the proper folding or assembly of the catalytic domain or alter protease substrate recognition and binding [3].

*TMPRSS3* mutations can be classified as mild or severe, and the hearing phenotype is dependent on the combination of the two *TMPRSS3* mutant alleles. Compound heterozygosity for a mild and severe mutation leads to postlingual hearing loss (DFNB8), whereas the combination of two severe mutations leads to profound hearing impairment with prelingual onset (DFNB10) [4]. Apparently, genotype-phenotype correlations can be drawn based on the position or the truncating/nontruncating nature of the *TMPRSS3* mutations.

Our study expanded the mutation spectrum of *TMPRSS3*.  Table 3 summarizes the type, position, origin, and mutation classification of the 39 *TMPRSS3* mutations reported to date, which are associated with ARNSHL in more than 15 ethnic groups worldwide. Eleven mutations were truncating and were predicted to lead to a prematurely terminated protein product or to nonsense-mediated decay of the mRNA, while another 28 *TMPRSS3* mutations were missense mutations leading to single amino acid substitutions. Almost all of the mutations were predicted to disrupt the proteolytic activity of the protein. The hearing impairment in these families was prelingual or postlingual, mostly with a typical ski-slope audiogram configuration. Consistent with previous reports, the *TMPRSS3* mutations identified in this study were associated with progressive hearing loss with considerable variability in the age of onset and degree of severity and this variability in hearing phenotype was both interfamilial and intrafamilial. The mutation summary data (Table 3) shows that not only protein-truncating mutations (frameshift, stop codon, and splice site mutations) but also missense mutations, particularly those located within the catalytic serine protease domain or close to the active site, have severe effects.

We observed that four combinations of *TMPRSS3* mutations resulted in prelingual, profound hearing impairment: c.1250G>A (p.Gly417Glu) and c.916G>A (p.Ala306Thr), c.1204G>A (p.Gly402Arg) and c.916G>A (p.Ala306Thr), c.809T>A (p.Ile270Asn) and c.916G>A (p.Ala306Thr), and c.1151T>G (p.Met384Arg) and c.1244T>C (p.Leu415Ser). The combination of c.323-6G>A and c.916G>A (p.Ala306Thr) was manifested by postlingual, milder hearing impairment. Our data suggest that the five novel missense mutations identified in this study have relatively severe effects. *TMPRSS3* c.916G>A (p.Ala306Thr), which was identified in five families from this study and one family from a previous study, is a pathogenic mutation in German, Dutch, and Korean deaf patients [4, 10, 11, 21], indicating that this mutation is the main contributor to the DFNB8/DFNB10 phenotype in many ethnicities. The proposal by Chung et al. that p.Ala306Thr could be a “founder mutation” was supported by the observation of linked haplotypes of STR markers segregating with hearing loss in two families [11]. Our haplotype analysis of six families showed linkage disequilibrium in four of them. Therefore, we propose that *TMPRSS3* c.916G>A (p.Ala306Thr) is likely to be a founder mutation in the Chinese population. Combining the data from this study and our previous work, we determined that mutations in *TMPRSS3* are a pathogenic cause of deafness in 7 of 151 (4.6%) Chinese families with ARNSHL. To the best of our knowledge, this is the first study to investigate the etiological contribution of *TMPRSS3* to deafness in a Chinese population.

This study had two minor limitations. First, there are no precise criteria for classifying mutations as severe or mild; therefore, we drew our own conclusions based only on the age of onset and severity of hearing loss. Second, we did not test for copy number variants in the samples; this remains an area for a future study.

In summary, combined with our previous study, we have described the clinical and genetic characteristics of seven Chinese families with ARNSHL carrying causative *TMPRSS3* mutations, resulting in the *TMPRSS3* mutation spectrum to be reported in a Chinese ARNSHL population for the first time. This should have an important impact on clinical patient management, genetic counseling, molecular diagnosis, and the development of advanced therapeutic strategies.

## Figures and Tables

**Table 1:** Distance of STR marker to TMPRSS3 Ala306.

STR marker	Distance to A306
D21S266	1,117,653
D21S1260	1,006,184
D21S266	312,954
Ala306	0
D21S1260	97,528
D21S266	358,798

**Table 2:** Clinical features and *TMPRSS3* mutation combinations of affected family members identified in the present study and our previous study.

Family number	Mutation 1	Mutation 2	Patient number	Phenotype	Age of onset	Progression	Vertigo
FH1523	c.36delC (p.Pro12fs)	c.916G>A (p.Ala306Thr)	1	Downsloping audiogram configuration with impairment of the low frequencies except 125 Hz at 3 years of age	Newborn	Yes	No
	c.316C>T (p.Arg106Cys)	c.916G>A (p.Ala306Thr)	5	Downsloping audiogram configuration with normal threshold of the low frequencies at 50 years of age Flat audiogram configuration with thresholds of about 80 dB at over 60 years of age	20–30 yo	Yes	No
6519	—	c.916G>A (p.Ala306Thr)	1	Moderate slope audiogram configuration with normal threshold of 250 Hz at 3 years of age	3 yo	Yes	No
6932	c.323-6G>A	c.916G>A (p.Ala306Thr)	1	Downsloping audiogram configuration with normal hearing threshold of 125 Hz and 256 Hz at 14 years of age	9 yo	Yes	No
8082	c.809T>A (p.Ile270Asn)	c.916G>A (p.Ala306Thr)	1	Downsloping audiogram configuration with impairment of the low frequencies at a very young age	3 yo	Yes	No
10706	c.1250G>A (p.Gly417Glu)	c.916G>A (p.Ala306Thr)	2	Flat audiogram configuration with thresholds of about 90 dB at 8 years of age	2 yo	Yes	No
8961	c.1204G>A (p.Gly402Arg)	c.916G>A (p.Ala306Thr)	1	Flat audiogram configuration with average PTA is are over 90 dB at 6 yo	2 yo	Yes	No
M234	c.1151T>G (p.Met384Arg)	c.1244T>C (p.Leu415Ser)	2	Flat audiogram configuration with average PTA is over 90 dB at 27 yo Downsloping audiogram configuration with impairment of the low frequencies at 35 yo	3 yo	Yes	No

**Table 3:** Overview of *TMPRSS3* mutations described in DFNA8/10, including those identified in the present study.

Mutation	Protein change	Exon	Domain	Origin	Mutation classification	Reference
c.323-6G>A		Intron4	LDLRA	Chinese Korean	Mild	Present study Ahmed et al., 2004 Weegerink et al., 2011
c.809T>A	p.Ile270Asn	E9	Serine protease	Chinese	Severe	Present study
c.916G>A	p.Ala306Thr	E9	Serine protease	Chinese German Korean Dutch	Severe	Present study Gao et al., 2017 Chung et al., 2014 Lee et al., 2013 Weegerink et al., 2011
c.1151T>G	p.Met384Arg	E11	Serine protease	Chinese	Severe	Present study
c.1204G>A	p.Gly402Arg	E12	Serine protease	Chinese	Severe	Present study
c.1244T>C	p.Leu415Ser	E12	Serine protease	Chinese	Severe	Present study
c.1250G>A	p.Gly417Glu	E12	Serine protease	Chinese	Severe	Present study
c.36delC	p.Pro12fs	E2	TM	Chinese	Severe	Gao et al., 2017
c.36dupC	p.Phe13fs	E2	TM	Turkish	N/A	Diaz-Horta et al., 2011
c.208delC	p.Thr70fs	E4	LDLRA	Pakistani Spanish Greek Newfoundlander	Severe	Ahmed et al., 2004 Weegerink et al., 2011 Battelino et al., 2016
c.268G>A	p.Ala90Thr	E4	LDLRA	UK Caucasian Moroccan	N/A	Charif et al., 2012
c.296C>A	p.Ser99X	E4	LDLRA	Chinese	Severe	Gu et al., 2014
c.308A>G	p.Asp103Gly	E4	LDLRA	Greek	N/A	Wattenhofer et al., 2002
c.310G>A	p.Glu104Lys	E4	LDLRA	Pakistani	N/A	Lee et al., 2012
c.310G>T	p.Glu104X	E4	LDLRA	Pakistani	N/A	Lee et al., 2012
c.316C>T	p.Arg106Cys	E4	LDLRA	Chinese Japanese	Mild	Gao et al., 2017 Miyagawa et al., 2013
c.325C>T	p.Arg109Trp	E5	SRCR	Pakistani Korean	Severe	Ahmed et al., 2004 Ben-Yosef et al., 2001
c.326G>A	p.Arg109Gln	E5	SRCR	Chinese	Mild	Gu et al., 2014
c.413C>G	p.Ala138Glu	E5	SRCR	British Korean	Mild	Weegerink et al., 2011
c.581G>T	p.Cys194Phe	E6	SRCR	Pakistani	Severe	Ahmed et al., 2004 Ben-Yosef et al., 2001
c.595G>A	p.Val199Met	E6	SRCR	Dutch Korean	Severe	Weegerink et al., 2011
c.607C>T	p.Gln203X	E6	SRCR	Japanese	Severe	Miyagawa et al., 2013
c.646C>T	p.Arg216Cys	E8	SRCR	German	Mild	Elbracht et al., 2007
c.647G>T	p.Arg216Leu	E8	SRCR	Turkish	Severe	Wattenhofer et al., 2005
c.726C>G	p.Cys242Trp	E8	SRCR	Pakistani	Severe	Shafique et al., 2014
c.743C>T	p.Thr248Met	E8	SRCR	Korean	Mild	Chung et al., 2014
c.753G>C	p.Trp251Cys	E8	SRCR	Tunisian	Severe	Masmoudi et al., 2001
c.767C>T	p.Arg256Val	E8	SRCR	Pakistani	N/A	Lee et al., 2012
c.782+8insT		Intron8	SRCR	Newfoundlander	Severe	Ahmed et al., 2004
c.988delA	p.Glu330fs	E10	Serine protease	Pakistani	Severe	Walsh et al., 2005
c.1019C>G	p.Thr340Arg	E10	Serine protease	Italian	Severe	Vozzi et al., 2014
c.1159G>A	p.Ala387Thr	E11	Serine protease	Japanese	Mild	Miyagawa et al., 2013
c.1180_1187del8ins68		E11	Serine protease	Palestinian	Severe	Scott et al., 2001
c.1192C>T	p.Gln398X	E12	Serine protease	Turkish	Severe	Wattenhofer et al., 2005
c.1211C>T	p.Pro404Leu	E12	Serine protease	Tunisian	Severe	Wattenhofer et al., 2005 Masmoudi et al., 2001
c.1219T>C	p.Cys407Arg	E12	Serine protease	Pakistani	Severe	Ahmed et al., 2004 Ben-Yosef et al., 2001 Lee et al., 2012
c.1273T>C	p.Cys425Arg	E12	Serine protease	Pakistani	N/A	Lee et al., 2012
c.1276G>A	p.Ala426Thr	E12	Serine protease	Dutch	Mild	Weegerink et al., 2011
c.1291C>T	p.Pro431Ser	E12	Serine protease	Italian	Severe	Vozzi et al., 2014

N/A: not available.

## Additional Points

The English in this document has been checked by at least two professional editors, both native speakers of English. For a certificate, please see http://www.textcheck.com/certificate/33mfci.
